# Screening for psychiatric morbidity in patients with advanced breast cancer: validation of two self-report questionnaires.

**DOI:** 10.1038/bjc.1991.305

**Published:** 1991-08

**Authors:** P. Hopwood, A. Howell, P. Maguire

**Affiliations:** Cancer Research Campaign Psychological Medicine Group, Christie Hospital and Holt Radium Institute, Withington, Manchester, UK.

## Abstract

Eighty-one patients with advanced breast cancer completed the Hospital Anxiety and Depression Scale (HADS) and Rotterdam Symptom Checklist (RSCL) to determine how well these questionnaires identified patients suffering from an anxiety state or depressive illness, compared with an independent interview by a psychiatrist who used the Clinical Interview Schedule. A threshold score was defined for each questionnaire which gave the optimal sensitivity and specificity. Seventy-five per cent of patients were correctly identified as suffering from an affective disorder by both the Rotterdam Symptom Checklist and by the Hospital Anxiety and Depression Scale. Twenty-one per cent of 'normal' patients were misclassified by the Rotterdam Checklist and 26% by the Hospital Anxiety and Depression Scale. When the HADs anxiety and depression subscales were analysed separately, the performance of the anxiety items was superior to that of the depression items. Both questionnaires were found to have good predictive value and could be used in patients with advanced cancer to help screen out those with an affective disorder.


*Br. J. Cancer* (1991), **64**, 353–356 © Macmillan Press Ltd., 1991

### Screening for psychiatric morbidity in patients with advanced breast cancer: validation of two self-report questionnaires

P. Hopwood[1], A. Howell[2] & P. Maguire[1]

[1]*Cancer Research Campaign Psychological Medicine Group;* [2]*Cancer Research Campaign Department of Medical Oncology, Christie Hospital and Holt Radium Institute, Wilmslow Road, Withington, Manchester M20 9BX, UK.*

**Summary** Eighty-one patients with advanced breast cancer completed the Hospital Anxiety and Depression Scale (HADS) and Rotterdam Symptom Checklist (RSCL) to determine how well these questionnaires identified patients suffering from an anxiety state or depressive illness, compared with an independent interview by a psychiatrist who used the Clinical Interview Schedule. A threshold score was defined for each questionnaire which gave the optimal sensitivity and specificity. Seventy-five per cent of patients were correctly identified as suffering from an affective disorder by both the Rotterdam Symptom Checklist and by the Hospital Anxiety and Depression Scale. Twenty-one per cent of 'normal' patients were misclassified by the Rotterdam Checklist and 26% by the Hospital Anxiety and Depression Scale. When the HADs anxiety and depression subscales were analysed separately, the performance of the anxiety items was superior to that of the depression items. Both questionnaires were found to have good predictive value and could be used in patients with advanced cancer to help screen out those with an affective disorder.

Clinicians treating women with advanced breast cancer have become increasingly concerned about their quality of life (Baum *et al.*, 1980; Bell *et al.*, 1985; Brinkely, 1985; Coates *et al.*, 1987; Gough *et al.*, 1983; Tannock *et al.*, 1988), yet, the recognition of psychological distress is handicapped by patients' unwillingness to disclose any emotional problems and doctors' and nurses' reluctance to enquire (Maguire *et al.*, 1980). Ways of improving the identification of psychological morbidity need to be found and a self-assessment approach warrants evaluation. Two self-report questionnaires, The Hospital Anxiety and Depression Scale (HADS) (Zigmond & Snaith, 1983) and The Rotterdam Symptom Checklist (RSCL) (de Haes *et al.*, 1990), seemed promising since they were both designed for use with physically ill patients. However, there is little published data concerning their performance in homogeneous cancer groups.

A study was conducted to determine how well these two questionnaires identified depressive illness and anxiety states in patients with advanced cancer of the breast.

### Patients and methods

Patients with advanced cancer of the breast attending medical oncology outpatient clinics over a 12 month period were included, providing they were free from dementia or cerebral metastases.

A research nurse explained the study and asked each patient to complete the two questionnaires. The HADS is designed to discriminate between anxiety and depression and is made up of a 7-item anxiety subscale and a 7-item depression subscale. Each item (for example, 'I can laugh and see the funny side of things') is rated on a 4-point scale (e.g. as much as I always do (0); not quite so much (1); definitely not so much (2); and not at all (3), giving maximum subscale scores of 21 for depression and anxiety respectively. The questionnaire assessed symptoms over the preceding week. The authors of the HADS suggested that scores $>11$ on either subscale were indicative of a case of depression or a case of clinical anxiety, whilst subscale scores in the 8–10 range represented borderline cases.

Received 16 February 1990; and in revised form 2 February 1991.

The RSCL contains three subscales: physical symptomatology due to disease and/or treatment (22 items); psychological symptoms (8 items) and activities of daily living (8 items). All items are rated on a 4-point scale (e.g. 'I feel tense'; not at all (0); a little (1); somewhat (2); very much (3)). The psychological subscale yields a maximum score of 24. The questionnaire assessed symptoms over the preceding 3 days.

The order in which these questionnaires were completed by patients was balanced so that they were equally represented in first and second positions. Their presentation was therefore alternated as each consecutive clinical session in which patients were being recruited.

Consecutive patients with a high score on either questionnaire were asked if they were willing to be interviewed for the research by the psychiatrist. With respect to the HADS, 'high scorers' were those patients with scores at or above the recommended threshold value of 11, on either subscale. The same value for the psychological subscale of the RSCL was selected following discussion with one of its authors (Pruyn, personal communication, 1983). In addition to patients with a high score, some patients with a low questionnaire score were also approached. This normally involved asking the next low scoring patient following a high scorer. Thirty-four patients had high scores on the HADS and 47 patients had low scores. Using the RSCL, 27 patients had scores $\geqslant 11$ on the psychological complaints subscale and 54 had low scored (i.e. $<10$). In total 44 patients out of the 81 recruited had a high score on either the HADS (17) or the RSCL (10) or both (17). Thirty-seven patients had low scores on both questionnaires. The interviews were conducted by a clinical psychiatrist (PH), who was blind to the questionnaire scores, using the Clinical Interview Schedule (Goldberg *et al.*, 1970) with additional questions in order to apply standardised psychiatric diagnostic criteria (DSM III) (American Psychiatric Association, 1980).

A diagnosis of depressive illness required the presence of depressed mood for a minimum of 4 weeks, plus at least four of the following symptoms: change in appetite or weight; insomnia or hypersomnia; psychomotor agitation or retardation; loss of interest including loss of libido; worthlessness or guilt; diminished concentration or suidical ideas. The diagnosis of an anxiety state required the presence of generalised persistent anxiety of at least one month's duration, together with symptoms from three of four categories: motor tension (for example, shakiness, tension, fidgeting); autonomic hyperactivity (for example, dizziness, paraesthesia, diarrhoea); apprehensive expectation (for example, worry, fear, anxious foreboding); and vigilance and scanning (for example, distractibility, poor concentration and irritability).

Where physical symptoms were clearly attributable to disease or treatment (for example, fatigue) they were ignored.

Patients found to have symptoms of depression or anxiety, but insufficient to meet these DSM III criteria were designated 'borderline' cases.

### Results

Two hundred and four patients completed the two questionnaires and 18 patients refused, giving a compliance rate of 92%. Eight-one patients agreed to be interviewed and there were no refusals.

### *Treatment*

Of the 81 patients interviewed 37 were receiving endocrine therapy, 24 were receiving chemotherapy, seven were taking corticosteroids and 13 were not receiving treatment.

### *Psychiatric morbidity*

Twenty (25%) patients were found at interview to have a depressive illness and/or anxiety state while 11 (14%) patients had a borderline mood disorder.

### *Performance of the two questionnaires in the study sample*

The sensitivity and specificity were calculated for the HADS anxiety and depression subscales and the RSCL psychological complaints subscale, using both the recommended threshold score (11) and a range of alternative cut-off scores. Sensitivity indicated the proportion of correctly identified cases (number of true cases/number of true cases plus number of false negatives) and hence the rate of false negatives. Specificity indicates the proportion of correctly identified non-cases (number of true non-cases/number of true non-cases plus number of false positives) and hence the false positive rate (1-specificity). The misclassification rate is calculated from the ratio false positives + false negatives/total number of sample.

Table I shows the performance of the two questionnaires based on the sample interviewed. Optimum sensitivity and specificity was found with a cut off value of 11 for each scale. The raw data used to calculate these values are shown in Table II. The sensitivity and specificity of each questionnaire were comparable and in an acceptable range. The misclassification rate for the HADS anxiety subscale (12%), was considerably lower than that for the depression subscale (25%).

An analysis was also carried out on the HADS using combined subscale scores to compare its performance as a unitary 14 item scale. The best cut-off score was 18, for which the sensitivity was 75%, specificity 74% and misclassification rate 26% (see Table III).

### *Performance of the two questionnaires in true community conditions*

Sensitivity and specificity are dependent on the ratio of high and low scores in the sample. We deliberatedly balanced this ratio in approximately equal proportions for the interview study, in order to include as many potential 'cases' as possible for the validation exercise. The outpatient population, from which the study sample was drawn, included more low scorers (133, 65%) than the interviewed sample (46%). Similarly the prevalence of high scorers was lower in the complete sample (71, 35%) than in the interviewed group (54%). It was desirable, therefore, to re-calculate the proportion of 'cases' to 'non-cases' in the interviewed sample to reflect the prevalence of high scorers and low scorers in the 204 outpatient attenders who completed both questionnaires.

Sensitivity and specificity values were then recalculated using these 'weighted' values for cases and non-cases, the results are shown in Table IV. The combined HADS subscales were used for this part of the analysis. Whilst the specificity remained relatively unchanged, the sensitivity of the HADS improved from 75% to 81% and that of the RSCL fell from 75% to 71%. The prevalence of probable cases in the whole sample (204) was calculated using the formula

$$\frac{\text{'weighted' no. cases} \times 100}{\text{total no. patients screened}} \qquad \frac{34.71 \times 100}{204}$$

The estimated prevalence for the whole sample screened was 17%.

### *Predictive values*

The accuracy of a screening instrument is also dependent on its positive predictive value (PPV) (Vecchio, 1966), that is the

**Table I** Sensitivity, specificity and misclassification rate for the RSCL psychological complaints subscale, HADS anxiety and depression subscales, based on the interviewed sample

| | *RSCL* | *HADS anxiety* | *HADS depression* |
|---|---|---|---|
| Sensitivity % | 75 | 75 | 75 |
| Specificity % | 80 | 90 | 75 |
| Misclassification rate % | 21 | 12.3 | 24.7 |

**Table II** Ratio of cases to non-cases, identified by clinical interview, at the optimum threshold value for each questionnaire

| | *Psychiatric assessment* | |
|---|---|---|
| | *Non-cases* | *Cases* |
| *(i) HADS depression* | | |
| *Scores* | | |
| 0–10 | 49 | 4 |
| 11+ | 16 | 12 |
| *(ii) HADS anxiety* | | |
| *Scores* | | |
| 0–10 | 62 | 3 |
| 11+ | 7 | 9 |
| *(iii) RSCL Psychological complaints* | | |
| *Scores* | | |
| 0–10 | 49 | 5 |
| 11+ | 12 | 15 |

**Table III** Ratio of cases to non-cases as identified by clinical interview, using a cut-off score of 18 for the HADS questionnaire (anxiety and depression scores combined)

| | *Psychiatric assessment* | |
|---|---|---|
| | *Non-cases* | *Cases* |
| *Scores* | | |
| 0–17 | 45 | 5 |
| 18+ | 16 | 15 |

Sensitivity = 75%; Specificity = 74%.

**Table IV** 'Weighted' data used to calculate the sensitivity and specificity of the HADS and RSCL questionnaires in true outpatient conditions

| | *Psychiatric assessment* | |
|---|---|---|
| | *Non-cases* | *Cases* |
| *(i) HADS* | | |
| Low scorers | 140.30 | 6.70 |
| High scorers | 28.98 | 28.01 |
| Sensitivity 80.7%; Specificity 88.88%. | | |
| *(ii) RSCL* | | |
| Low scorers | 150.06 | 9.22 |
| High scorers | 19.22 | 24.77 |

Sensitivity 71.36%; Specificity 88.65%.

probability of a high score being a true psychiatric case. In clinical practice this value is important in determining the utility of an instrument, since it indicates the probability of detecting cases in a given population of patients. It is calculated from the ratio of the number of correctly identified cases/total number of persons with high scores. The PPV of a screening test is dependent on a prevalence in a given population and increases as prevalence rises. The PPV for the RSCL was 55.6%, which is high. For the HADS scale overall the value was 49.6% which is also very acceptable; the PPV for the anxiety subscale was 56.3% and that for the depression subscale 42.9%. (Using weighted data, the values for the two questionnaires were 56.3% and 49.2% respectively.) The negative predictive values (NPV) were also calculated, that is, the proportion of low scorers who are not cases. The value for the RSCL was 80.3% and that for the HADS 82.7%. In other words, both questionnaires used as screening instruments would correctly identify one in every two high scorers as a case and have a relatively low risk of misclassifying a low scorer as a case. The ability of the HADS to accurately detect anxiety cases is high but its performance to detect case depression more modest.

*Borderline affective disorder*

The HADS questionnaire has been used to discriminate patients with borderline depression and anxiety using scores in the 8–10 range (Zigmond & Snaith, 1983). Of the patients interviewed, three out of six patients with a diagnosis of borderline anxiety and three out of eight with borderline depression were misclassified as cases by the HADS using the range of scores suggested by Zigmond and Snaith. Further analysis of the HADS in the borderline range of scores is underway but will not be reported here.

### Discussion

The statistical analyses indicated that both questionnaires performed reasonably well and were suitable for use as screening instruments. However, both warrant refinement to improve their accuracy in detecting cases. Using the HADs as a 14-item scale, our results are very similar to those reported by Razavi *et al.* (1990), who suggested an optimal cut off point of 19 for major depressive disorder, associated with 70% sensitivity and 25% false positive rate. (Our cut off point of 19 gave exactly the same sensitivity and specificity values, but a threshold of 18 was superior from our data.) When using the separate subscales, the performance of the anxiety subscale was superior to that for depression, in terms of its positive predictive power and false positive rate. Our cut off value of 11 with the HADS depression subscale is a little higher than that reported by Razavi, but a lower value gave an unacceptable false positive rate (39%) with our data.

When screening for illness, it is desirable to achieve a 100% detection rate. Whilst this can be achieved with these questionnaires, it is only at the expense of including a high proportion of false positives, which would create an unacceptable interview load.

In clinical practice, patients with high scores warrant further assessment by a brief interview, as it is posisble to discriminate true cases from false positives on this basis. The 'optimal' sensitivity is aimed to identify an acceptable balance in terms of accuracy and clinical feasibility. An instrument with good predictive power is very valuable, since it will reduce the interview load. The RSCL was superior to the HADS in this respect.

Patients misclassified by both questionnaires are worth examining in greater detail. Of the five cases who were false negatives according to the RSCL, four were identified by the HADS, completed on the same occasion. Similarly, the RSCL identified two cases missed by the HADS, suggesting that the use of two questionnaires concurrently or sequentially may be of benefit. Other factors may have contributed to the misclassification rate: firstly the clinical 'case' definition may have been too stringent, and hence the cut off score set too high. Secondly, the omission of somatic items that were thought due to disease or treatment may have raised the threshold for the clinical diagnosis. Further work in much larger samples is currently underway to assess the importance of somatic items in the diagnosis of psychiatric morbidity in cancer patients. A current advantage in both questionnaires tested in this study is that they exclude somatic items from the psychological subscales.

Among the false positive scores were two patients with anxious personalities and two with bereavement reactions, and the questionnaires were not expected to discriminate such cases. Also included were patients with borderline depression or anxiety as judged by a clinical interview. Some of these patients would fulfill a DSM diagnosis of adjustment disorder (not used in this analysis). Such reactions may give rise to substantial psychological distress but this does not necessarily correlate with a formal psychiatric diagnosis of depressive illness or anxiety state.

The estimated prevalence of psychological illness, from the 'weighted' data, may be considered low at 17% and ideally the prevalence should be derived from an independent sample from the same population. However, this prevalence is double the psychological morbidity reported by Dean (1987) in a sample of patients with early breast cancer. Like Dean, standard methods for defining psychiatric illness were used in this study, and strict criteria for defining a case were applied. Derogatis *et al.* (1983) reported a prevalence rate for psychological morbidity of 47% in a heterogenous sample of cancer patients, but of these only 13% were ascribed as DSM III diagnosis of depressive disorder or anxiety state. Sixty-eight per cent of their diagnoses were deemed to be adjustment reactions.

It proved feasible to use both questionnaires in a busy clinical setting. The HADS has the advantage of being short, and of discriminating to an extent between cases of depression and anxiety. The RSCL has additional useful questions about other aspects of the patients' quality of life, namely physical symptoms, treatment toxicity and functional status. Other instruments have been produced specifically for use in cancer research or practice (Bell *et al.*, 1985; Coates *et al.*, 1987; Padilla *et al.*, 1981; Priestman & Baum, 1976; Selby *et al.*, 1984) but these have not been validated against a psychiatric interview. This makes their scores difficult to interpret.

The performance of these questionnaires is much superior to the detection rate of doctors and nurses involved in cancer care. Only 22% of patients with psychological problems following mastectomy are recognised by those concerned with their aftercare (Maguire *et al.*, 1980). However, Maguire found that specialist nurses trained to detect such problems, can identify up to 80% of patients with depression or an anxiety state. A two stage process of assessment could be used, in which patients are serially screened for psychiatric disorder using one of these questionnaires. Those with above threshold scores would then be assessed further by a specialist nurse. This would provide a practical way of identifying patients who might need help in the setting of a busy oncology clinic. Such screening questionnaires could also be used in clinical trials to measure the psychological dimension of quality of life.

In using either questionnaire as a screening instrument careful preparation is advisable: sensitivity, specificity and cut-off values should be checked, and the predictive value should be calculated according to the known prevalence of affective disorder in the population of patients to be screened. Used in this way, these two instruments will provide a valuable clinical tool in the detection of psychological morbidity.

This study was supported by Organon International BV, OS, Holland. We are most grateful for the help of research nurses; Mrs Anna Leah and Mrs Patricia Phillips and for advice concerning the analysis from Professor D.P. Goldberg.

### References

AMERICAN PSYCHIATRIC ASSOCIATION, COMMITTEE ON NOMENCLATURE AND STATISTICS (1980). *Diagnostic and Statistical Manual of Mental Disorders*. Washington DC. American Psychiatric Association.

BAUM, M., PRIESTMAN, T., WEST, R. & JONES, E. (1980). A comparison of subjective responses in a trial comparing endocrine with cytotoxic treatment in advanced carcinoma of the breast. *Eur. J. Cancer*, Suppl: 223.

BELL, D.R., TANNOCK, I.F. & BOYD, N.F. (1985). Quality of life measurement in breast cancer patients. *Br. J. Cancer*, **51**, 577.

BRINKLEY, D. (1985). Quality of life in cancer trials. *Br. Med. J.*, **291**, 685.

COATES, A., GEBSKI, V., STAT, M. & 14 others (1987). Improving the quality of life during chemotherapy for advanced breast cancer. *New Engl. J. Med.*, **317**, 1490.

DEAN, C. (1987). Psychiatric morbidity following mastectomy: preoperative predictors and types of illness. *J. Psychosom. Res.*, **31**, 385.

DEROGATIS, L.R., MORROW, G.R., FETTING, J. & 5 others (1983). The prevalence of psychiatric disorders among cancer patients. *JAMA*, **249**, 751.

GOLDBERG, D.P., COOPER, B., EASTWOOD, M.R., KEDWARD, H.B. & SHEPHERD, M. (1970). A standardised psychiatric interview for use in community surveys. *Br. J. Prev. Soc. Med.*, **24**, 18.

GOUGH, I.R., FURNIVAL, C.M., SCHILDER, L. & GROVE, W. (1983). Assessment of quality of life of patients with advanced cancer. *Eur. J. Clin. Oncol.*, **19**, 1161.

DE HAES, J.C.J.M., VAN KNIPPENBERG, F.C.E. & NEIJT, J.P. (1990). Measuring psychological and physical distress in cancer patients: structure and application of the Rotterdam Symptom Checklist. *Br. J. Cancer*, **62**, 1034.

MAGUIRE, G.P., TAIT, A., BROOKS, M., THOMAS, C. & SELLWOOD, R.S. (1980). Effect of counselling on the psychiatric morbidity associated with mastectomy. *Br. Med. J.*, **281**, 1454.

PADILLA, G., PRESANT, C.A., GRANT, M., BAER, C. & METTER, G. (1981). Assessment of quality of life in cancer patients. *Proc. Am. Assoc. Cancer Res. & Am. Soc. Clin. Oncol.*, **22**, 397. Abstract C-255.

PRIESTMAN, T.J. & BAUM, M. (1976). Evaluation of quality of life in patients receiving treatment for advanced breast cancer. *Lancet*, **i**, 899.

PRUYN, J.F.A. (1983). (Personal Communication).

RAZAVI, D., DELVAUX, N., FARVACQUES, C. & RABAYE, E. (1990). Screening for adjustment disorders and major depressive disorders in cancer in-patients. *Br. J. Psychiatr.*, **156**, 79.

SELBY, P.J., CHAPMAN, J.A.W., ETAXADI-AMOLI-J. DALLEY, D. & BOYD, N.F. (1984). The development of a method for assessing the quality of life of cancer patients. *Br. J. Cancer*, **50**, 13.

TANNOCK, I.F., BOYD, N.F., DE BOER, G. & 6 others (1988). A randomized trial of two dose levels of Cyclophosphamide, Methotrexate and Fluorouracil chemotherapy for patients with metastatic breast cancer. *J. Clin. Oncol.*, **6**, 1377.

VECCHIO, T.J. (1966). Predictive value of a single diagnostic test in unselected populations. *New Engl. J. Med.*, **274**, 1171.

ZIGMOND, A.S. & SNAITH, R.P. (1983). The Hospital Anxiety and Depression Scale. *Acta Psychiatr. Scand.*, **67**, 361.


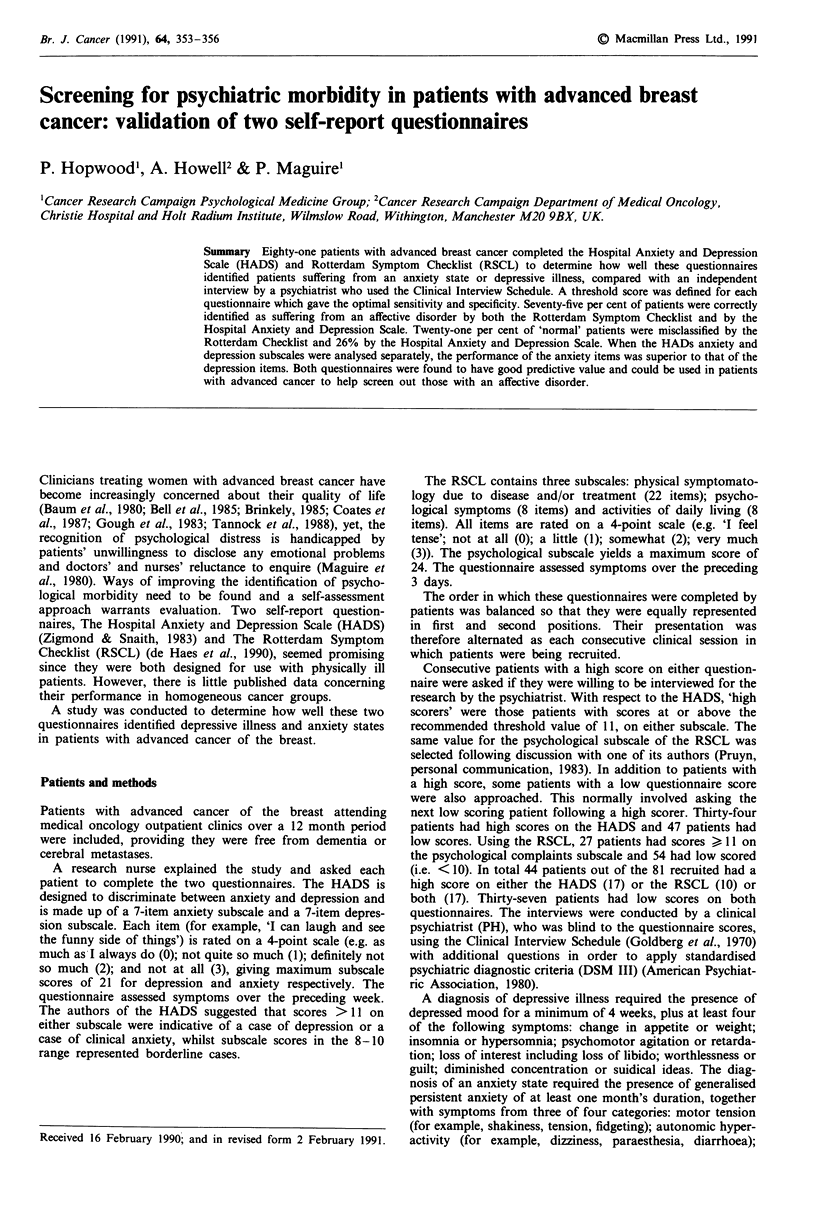

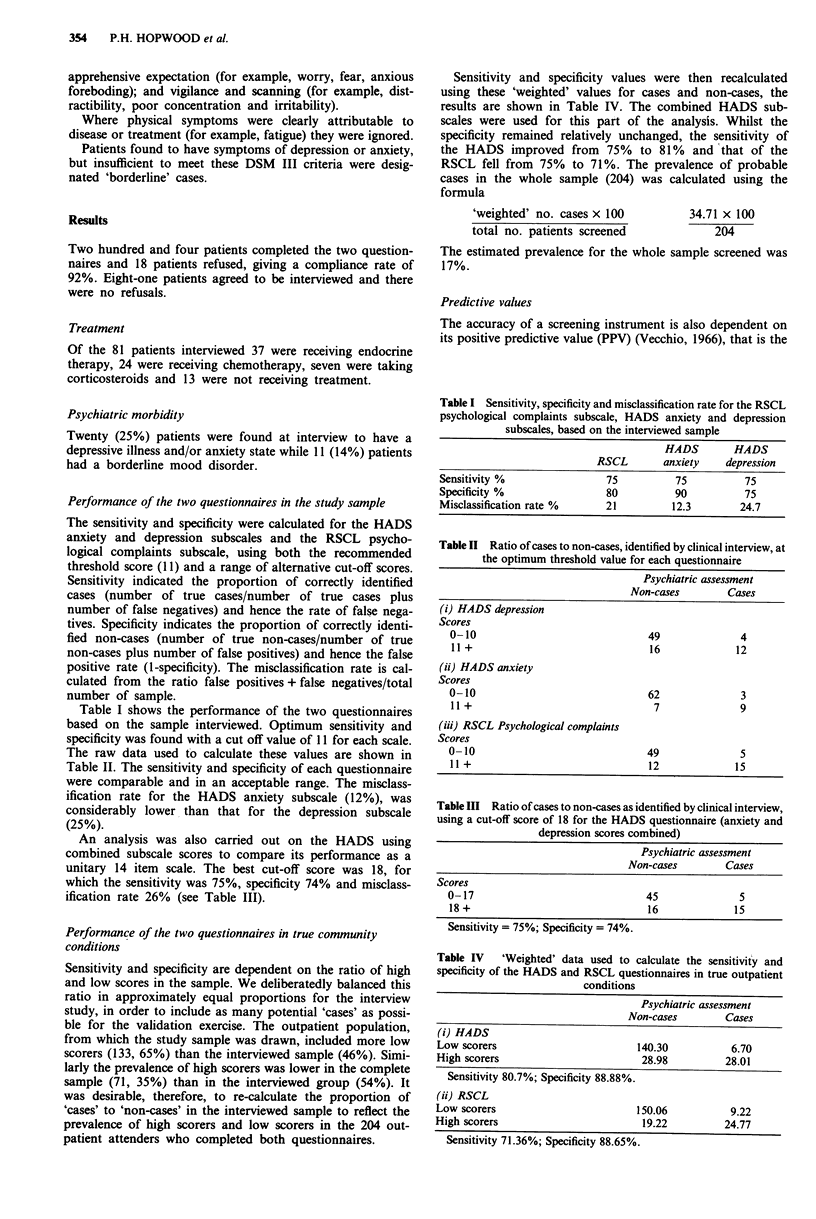

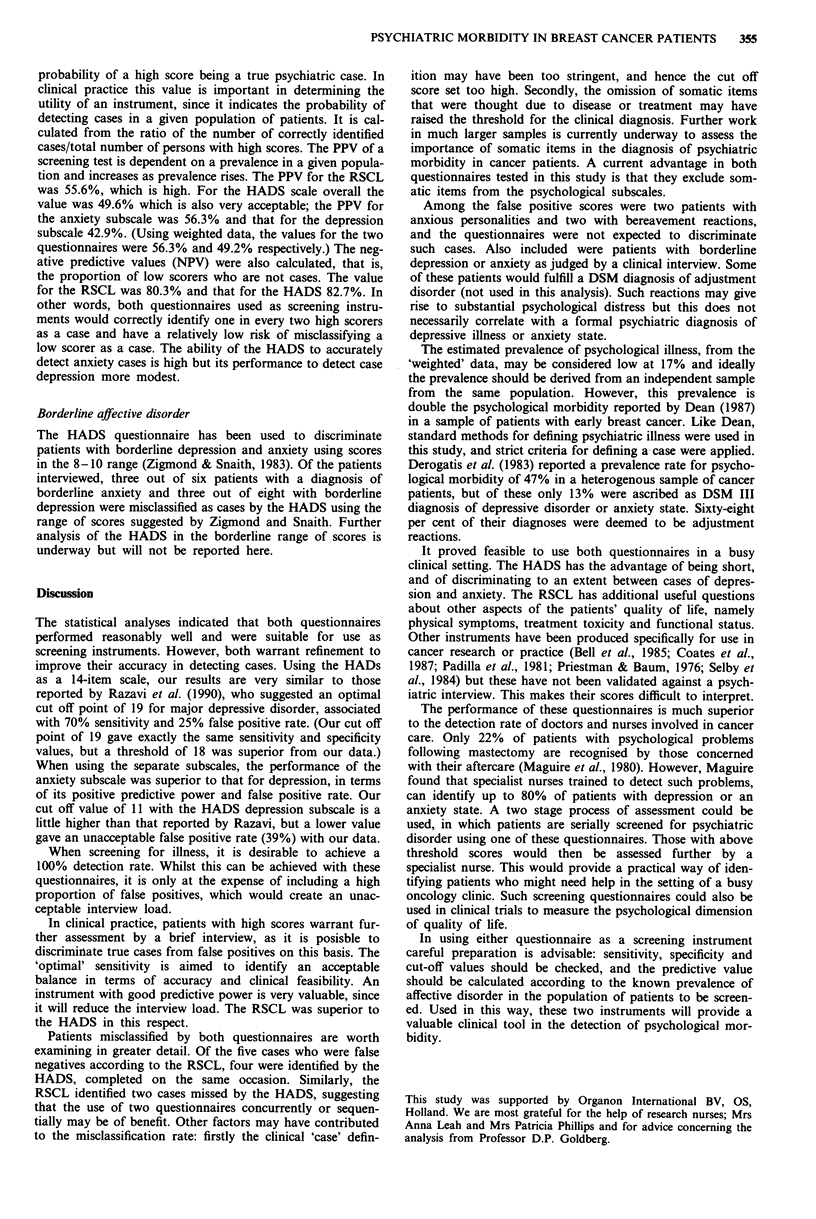

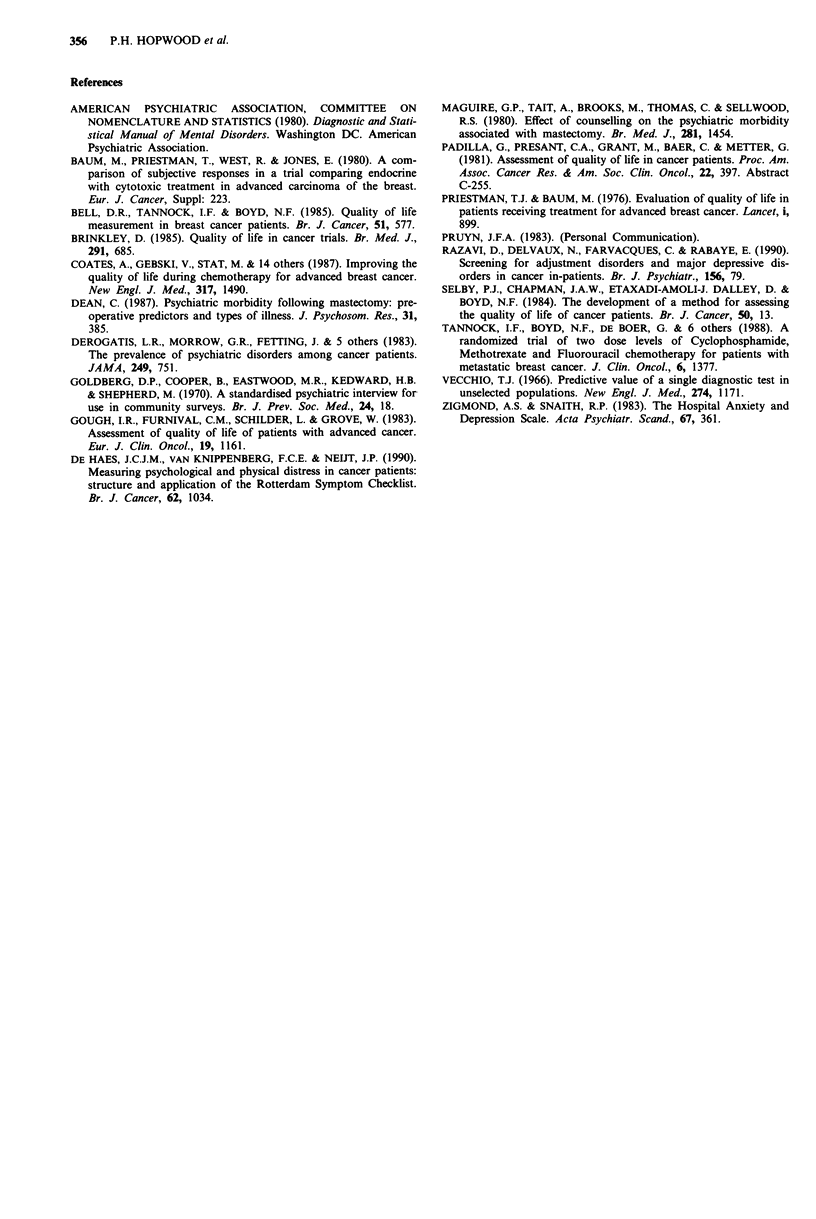


## References

[OCR_00466] Baum M., Priestman T., West R. R., Jones E. M. (1980). A comparison of subjective responses in a trial comparing endocrine with cytotoxic treatment in advanced carcinoma of the breast.. Eur J Cancer.

[OCR_00472] Bell D. R., Tannock I. F., Boyd N. F. (1985). Quality of life measurement in breast cancer patients.. Br J Cancer.

[OCR_00475] Brinkley D. (1985). Quality of life in cancer trials.. Br Med J (Clin Res Ed).

[OCR_00479] Coates A., Gebski V., Bishop J. F., Jeal P. N., Woods R. L., Snyder R., Tattersall M. H., Byrne M., Harvey V., Gill G. (1987). Improving the quality of life during chemotherapy for advanced breast cancer. A comparison of intermittent and continuous treatment strategies.. N Engl J Med.

[OCR_00484] Dean C. (1987). Psychiatric morbidity following mastectomy: preoperative predictors and types of illness.. J Psychosom Res.

[OCR_00489] Derogatis L. R., Morrow G. R., Fetting J., Penman D., Piasetsky S., Schmale A. M., Henrichs M., Carnicke C. L. (1983). The prevalence of psychiatric disorders among cancer patients.. JAMA.

[OCR_00494] Goldberg D. P., Cooper B., Eastwood M. R., Kedward H. B., Shepherd M. (1970). A standardized psychiatric interview for use in community surveys.. Br J Prev Soc Med.

[OCR_00499] Gough I. R., Furnival C. M., Schilder L., Grove W. (1983). Assessment of the quality of life of patients with advanced cancer.. Eur J Cancer Clin Oncol.

[OCR_00510] Maguire P., Tait A., Brooke M., Thomas C., Sellwood R. (1980). Effect of counselling on the psychiatric morbidity associated with mastectomy.. Br Med J.

[OCR_00521] Priestman T. J., Baum M. (1976). Evaluation of quality of life in patients receiving treatment for advanced breast cancer.. Lancet.

[OCR_00528] Razavi D., Delvaux N., Farvacques C., Robaye E. (1990). Screening for adjustment disorders and major depressive disorders in cancer in-patients.. Br J Psychiatry.

[OCR_00535] Selby P. J., Chapman J. A., Etazadi-Amoli J., Dalley D., Boyd N. F. (1984). The development of a method for assessing the quality of life of cancer patients.. Br J Cancer.

[OCR_00538] Tannock I. F., Boyd N. F., DeBoer G., Erlichman C., Fine S., Larocque G., Mayers C., Perrault D., Sutherland H. (1988). A randomized trial of two dose levels of cyclophosphamide, methotrexate, and fluorouracil chemotherapy for patients with metastatic breast cancer.. J Clin Oncol.

[OCR_00544] Vecchio T. J. (1966). Predictive value of a single diagnostic test in unselected populations.. N Engl J Med.

[OCR_00548] Zigmond A. S., Snaith R. P. (1983). The hospital anxiety and depression scale.. Acta Psychiatr Scand.

[OCR_00504] de Haes J. C., van Knippenberg F. C., Neijt J. P. (1990). Measuring psychological and physical distress in cancer patients: structure and application of the Rotterdam Symptom Checklist.. Br J Cancer.

